# Subjective speech quality measurement with and without parallel task: Laboratory test results comparison

**DOI:** 10.1371/journal.pone.0199787

**Published:** 2018-07-02

**Authors:** Hakob Avetisyan, Jan Holub

**Affiliations:** Department of Measurement, Faculty of Electrical Engineering, Czech Technical University, Prague, Czech Republic; University of York, UNITED KINGDOM

## Abstract

This paper focuses on a novel methodology of subjective speech quality measurement and repeatability of its results between laboratory conditions and simulated environmental conditions. A single set of speech samples was distorted by various background noises and low bit-rate coding techniques. This study aimed to compare results of subjective speech quality tests with and without a parallel task deploying the ITU-T P.835 methodology. Afterward, tests results performed with and without a parallel task were compared using Pearson correlation, CI95, and numbers of opposite pair-wise comparisons. The tests show differences in results in the case of a parallel task.

## Introduction

Each generation of mobile phones has different advanced features and characteristics designed to have a better quality of voice processing and noise suppression. For this purpose, various subjective and objective tests are performed to analyze, compare and improve the audio quality emerging mobile technologies. Subjective speech quality testing is designed for collecting subjective opinions from human test subjects deploying standardized procedures as specified, e.g., in [1]. Objective methods [2] are used to replace test subjects using psycho-acoustic modeling, comparing clean and distorted speech samples algorithmically. Outputs from these two method groups are often mapped to the subjective quality scale Mean Opinion Score (MOS) [2]. Comparing subjective and objective quality tests, subjective tests are believed to provide more accurate results but are also more demanding regarding time, equipment, effort and price. The main point, however, is the fundamental philosophy of currently used test methods: Test subjects are seated in anechoic or semi-anechoic test room and are fully focused on listening to the tested material. In real life, the users are usually performing multiple tasks at once (such as talking on the phone while working on PC, walking or even driving a car, or visually monitoring a screen where airplane location and approach situation is displayed while communicating on radio-link with the airplane pilot).

This paper deals with a novel technique of subjective testing with an implementation of a parallel task which simulates a real environmental situation. The reported experiment aims to verify if the ITU-T P.835 [3] methodology is suitable for parallel task incorporation, to identify potential differences in human perception under a parallel task situation and to demonstrate their impact to speech quality perception. Previously, comparisons between tests in laboratory conditions (without a parallel task) were performed, which didn’t show any crucial differences between tests performed in different laboratories [4–6].

The paper is structured as follows. After the Background section, the experiment description is given, providing information about methods, tested samples and equipment used. Next, we provide data analysis of measured speech quality, noise annoyance, and overall quality (as per ITU-T P.835 [3]) and compare results with and without a parallel task. Alongside Pearson correlation coefficient and CI95 uncertainty intervals, pairwise comparisons between each couple of tests are also provided. The final section contains conclusions and motivations for future work.

## Background

ITU-T Recommendation P.835 [3] describes methods for evaluating speech quality in noisy (and partially de-noised) speech. A typical example of its application is a comparison of different noise suppression algorithms. The P.835 methodology makes it possible to evaluate speech quality and noise levels separately. Test environment parameters are adopted from ITU-T P.800 [1]. Listeners evaluate tested samples on a five-point scale. This procedure is particularly suitable for samples processed by noise canceling algorithms that remove certain part of background noise but also corrupt the speech itself. Therefore, the principle of P.835 is to repeat the assessment of each speech sample three times, requiring the subjects to focus on a different aspect of the sample quality during each assessment. For the first half of samples, the subjects are asked to focus on speech quality only during the first playback, noise annoyance during the second playback and overall sample quality during the third (last) playback. For the second half of samples, subjects are asked to judge noise annoyance during the first playback, speech quality during the second playback and, identical to the first half of samples, overall sample quality for the third playback. The order of sample presentation is randomized.

The existing test methods and recommendations are based on the intuitive assumption [7] that the case of laboratory testing where the subjects are fully concentrated to the test procedure provides the most sensitive results compared to any real scenario when the users are distracted by performing other tasks. Multiple experiments that including a dual task have been performed on impaired [8] or child [9] subjects and do not focus on the average adult population. Subjects acquired from the common population are usually tested for a relationship between listening effort and dual task introduction [10–15].

## Experiment description

For data analysis, two subjective tests were held in subjective testing laboratory based in Prague, Czech Republic. They are named as A and B. Test A was performed in July 2015 and test B in January 2017. Test subjects from test A were different from test B. Test A contained 32 subjects and test B included 25 subjects. The test subjects were hired by professional listening lab service using social media advertisements. A mixture of subjects’ nationalities has been used (American, British, German, French, Czech, and Slovak). The exact nationality distribution is shown in Table 1. The English language proficiency of non-English participants was higher than average as verified by a short written English quiz, preceding the subjective testing. The written quiz was selected due to its short duration; despite the fact it is not an optimal means of assessing the ability to understand the spoken language. However, language understanding is not a necessary condition for speech quality assessment as demonstrated in [16]. The gender structure of the listening panels was balanced–test A included 16 male and 16 female test subjects while test B included 13 male and 12 female subjects. The age distribution approximately followed human population age distribution in the range between 18 and 65 years of age (average age: 28,4).

**Table 1:** Nationality distribution in tests A and B.

	U.S.	British	German	French	Czech	Slovak	TOTAL
**Test A**	2	2	3	4	15	6	**32**
**Test B**	1	2	2	3	12	5	**25**

A single English sample set was used in both experiments. The speech sample set was prepared following requirements of [1] and [3]. Original studio recordings were spoken by native professional English speakers (two male, two female voices). A selection of Harvard phonetically balanced sentences from the Appendix of IEEE Subcommittee on Subjective Measurements was used. Contemporary coders AMR WB [17] and EVS [18] and selected cases of background noise (Cafeteria, Mensa, Road, Pub, Office, Car, all adopted from [19]) were used to create a balanced set of realistic speech samples that covered a full coverage of quality. The background noise was mixed with speech material following ITU-T P.835 [3] Appendix 1. The final sample selection contained 22 conditions. Table 2 details the samples used.

**Table 2:** Test sample conditions.

Sample type (coder, bit rate)	Noise type acc. to [19]	SNR (dB) acc. to [3]	Number of samples
AMR WB 12,65k	Cafeteria	14,8	8
AMR WB 12,65k	Mensa	19,5	8
AMR WB 12,65k	Road	7,9	8
AMR WB 12,65k	Pub	8,4	8
AMR WB 12,65k	Office	25,3	8
EVS WB 13,2k	Cafeteria	14,8	8
EVS WB 13,2k	Mensa	19,5	8
EVS WB 13,2k	Road	7,9	8
EVS WB 13,2k	Pub	8,4	8
EVS WB 13,2k	Office	25,3	8
Reference 1–5	n/a	n/a	10
Reference 6,10	Car	0	4
Reference 7,11	Car	12	4
Reference 8,12	Car	24	4
Reference 9	Car	36	2

The test methodology was based on recommendation ITU-T P.835. As already discussed in the Background section, the concept of this standard is to make subjects listen to the same sample three times: first time for assessing the speech quality, second time–the noise annoyance, and the third time–the overall sample quality. As required by P.835, half of the test was performed in speech-noise-overall and the other half noise-speech-overall orders. MOS scores were obtained separately for Speech quality (S-MOS), Noise annoyance (N-MOS) and Overall sample quality (G-MOS). The terms S-MOS, N-MOS, and G-MOS, are adopted from ETSI TS 103 106 [20] and ETSI EG 202 396–3 [21]. These terms replace in the further text the original SIG, BAK, and OVRL ratings used in [3].

During test A, a simple P.835 test without any parallel task was performed. During test B, an additional parallel task was included to distract test subjects from fully concentrating on the subjective testing.

Both mental and physical parallel tasks are used in existing experiments [8–15]. To avoid the problem of generated load inequity for differently physically or mentally developed subjects, we designed a combined parallel task, incorporating both physical and mental efforts: A simple game deploying a professional laser shooting simulator (Simway) was used. Always a group of three subjects was evaluating the samples; however, at any given time one of them was a “shooter,” and other two were “counters.” The “shooter’s” task was to shoot as many in-game ducks as they could, and the “counters’” task was to count every single shot duck. The turn of the shooter was changed randomly using a light-bulb indicating who the current shooter was. The three bulbs (one in front of each subject) were operated by a random number generator always ensuring only one lamp was on, and every 40 seconds another lamp activated. The reason for swapping the roles was the shooting simulator limitation–only one single shooter is allowed at a time. Running the test separately for each subject, with each subject only as a shooter, would be extremely time-consuming. The compromising solution was to assign the “shooter” role randomly among three subjects, all of them assessing the speech samples in parallel. The samples were played out in random order using a different randomization for each listening panel.

## Materials and methods

Our experiment involved human participants and has been approved by Advisory Committee of the Dean of Faculty of Electrical Engineering, Czech Technical University in Prague, decision letter dated April 17th, 2015. All experiments were performed in accordance with the Declaration of Helsinki and relevant local guidelines and regulations. All involved subjects provided their written informed consent prior the experiment. There are no subject identifying details (HIPAA) in our contribution.

For the sound reproduction, Sennheiser HD 600 professional headphones were used. Votes were collected using a professional voting device. The used low-reverberation listening rooms conformed to requirements of [1]. Its reverberation time was 185ms and background noise level below 30dB SPL (A) without significant peaks in spectra.

All test results and their evaluation are available as supporting information files and also at protocols.io under dx.doi.org/10.17504/protocols.io.nwwdffe

## Results and data analysis

In S1, S2 and S3 Figs, the correlations between S-MOS, N-MOS, and G-MOS values are presented. The values are highly correlated. Nevertheless, there are interesting values worth mentioning.

Speech MOS (S-MOS) comparison between A and B tests are shown in S1 Fig. Its Pearson correlation coefficient value is 0.971. During the voting process of speech samples, the subjects voted on speech signal distortion (5 –not distorted to 1 –very distorted), as shown in Table 3.

**Table 3:** Test questions as per ITU-T P.835.

Opinion Score	Speech signal rating scale	Background noise rating scale	The overall quality rating scale
5	Not distorted	Not noticeable	Excellent
4	Slightly distorted	Slightly noticeable	Good
3	Somewhat distorted	Noticeable but not intrusive	Fair
2	Fairly distorted	Somewhat intrusive	Poor
1	Very distorted	Very intrusive	Bad

In the second part, the subjects were voting for background noise annoyance (5 –not noticeable to 1 –very intrusive). S2 Fig shows noise annoyance MOS correlations between A and B. Its Pearson correlation coefficient value is 0,982.

Finally, during the third part, the subjects were voting for the overall quality of each sample (5 –excellent to 1 –bad). For the second half of each experiment, the order of second and third voting was swapped as required by P.835. In S3 Fig, overall quality MOS correlations between A and B tests are shown. The Pearson correlation coefficient is 0.989.

In S1 Fig, there are two interesting points which do not correspond to overall results of the tests. The points are marked with red circles. Both points provide a similar evaluation in the A-tests (3.781 and 4.000) while in the B-tests their rank order is significantly opposite (4.417 and 3.417). By analysis of the sound files for the involved conditions we conclude that this order swapping is caused by voting mistakes caused by the introduction of the parallel task. The subjects were not able to distinguish properly between speech distortion and strong background noise. This means that some subjects decreased the speech quality score due to background noise even for non-distorted speech and also considered speech distorted by artificial coding artifact as noisy. It indicates that the P.835 methodology is too complex if used with the parallel task of the described type. Not all subjects can correctly assess speech distortion (only) and background noise annoyance (only) in different playouts as required by the P.835, as they are distracted by another task in parallel.

The graphs show that the subjects voted similarly. Correlation values are close to the maximum value of 1. However, as indicated in S1 Fig, certain sample pairs are ranked oppositely with and without a parallel task. For this purpose, pair-wise comparisons [22] were performed as described further.

### Pairwise comparison of each test

After the data correlations procedure, pairwise comparisons for the tests were evaluated. The comparison was performed in following way: First, global MOS values of the first test were compared with global MOS values of the second test. Afterward, the absolute difference between each pair of samples was calculated. There were 231 cases (22 datasets).

After the pairwise comparison between Global qualities (G-MOS), ten differences were found which is 4.3% of all cases. In these cases, users preferred one sample out of the pair without the parallel task but preferred the other one in the pair with the parallel task. Except for one case (the one marked by circles in S1 Fig and described in the section Results and Data analysis) statistical analysis has shown those differences are statistically significant only at a confidence level 0,2 (CI80) but statistically insignificant at a confidence level of 0,05 (CI95). More subjects would be needed to obtain statistically more significant data. Although, the single case mentioned above is significant at confidence level 0,05 (CI95).

Table 4 includes information about the average Confidence Intervals of each type of MOS for both tests. CI95 increases with parallel task introduction.

**Table 4:** Average CI95 of each test.

	Average CI95: S-MOS	Average CI95: N-MOS	Average CI95: G-MOS
A	0,133	0,117	0,113
B	0,155	0,137	0,148

## Conclusion and motivation for future work

A novel subjective testing methodology has been designed and demonstrated. The purpose of the parallel task during subjective testing was to bring the test results closer to realistic conditions. In total, 57 subjects participated in 2 different tests with and without implementation of the parallel task.

Pearson correlations between tests were calculated, and positions of values of subjects’ votes were plotted in graphs. Due to non-consistent values, pair-wise comparisons were performed, and ten differences were found.

Although the test results were highly correlated, certain conditions indicate different pair rankings after the parallel task is introduced. The resulting analysis indicated voting mistakes because of loss of subjects’ concentration due to parallel task introduction. Therefore, we conclude that ITU-T P.835 methodology is too complicated to be combined successfully with a complex parallel task as described here.

In the future, it is planned to continue the investigation, experimenting with less complex parallel task within P.835 context or using different methodology (e.g., ITU-T P.800) for the existing parallel task. Also, standardization effort will be initiated to define parallel task subjective testing as a logical counterpart to traditional laboratory subjective speech quality tests.

## Supporting information

S1 Fig **Speech MOS (S-MOS) of A and B tests.** Both axes have the values of MOS (1–5).
(TIF)

S2 Fig **Noise annoyance MOS (N-MOS) of A and B tests.** Both axes have the values of MOS (1–5).
(TIF)

S3 Fig **Overall quality MOS (G-MOS) of A and B test.** Both axes have the values of MOS (1–5).
(TIF)

S1 File Subjective data.xlsx. Detailed results of A and B experiments.
(XLSX)

S2 File Samples. Speech samples used for both the A and B experiments.
(ZIP)
