# Supplementary figures and images for: Subjective speech quality measurement with and without parallel task: Laboratory test results comparison
Source: PLoS One. 2018 Jul 2;13(7):e0199787. doi: 10.1371/journal.pone.0199787 (PMC6028083; doi:10.1371/journal.pone.0199787)

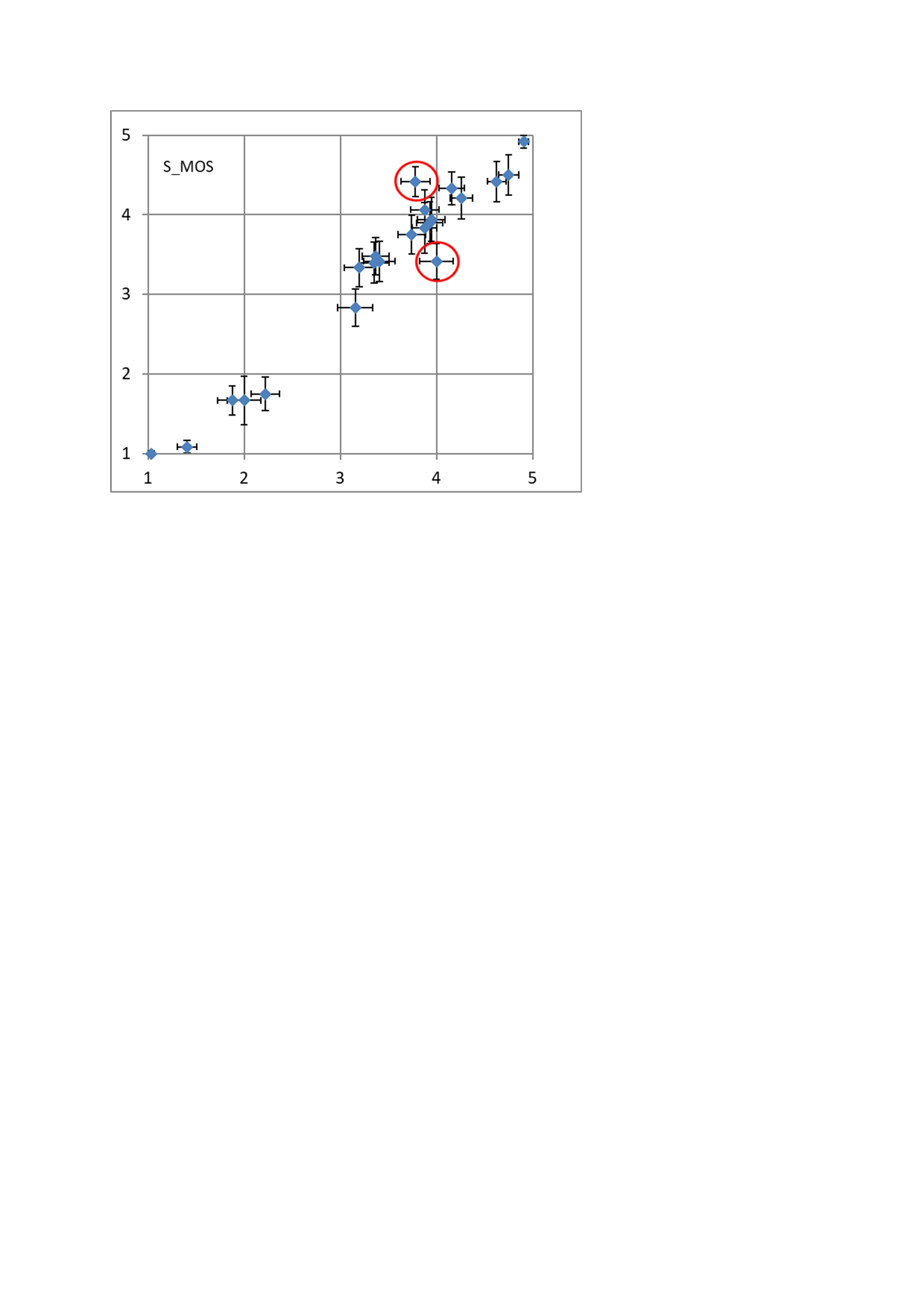

Supplement: S1 Fig — Speech MOS (S-MOS) of A and B tests. Both axes have the values of MOS (1–5). (TIF) [file pone.0199787.s001.tif]

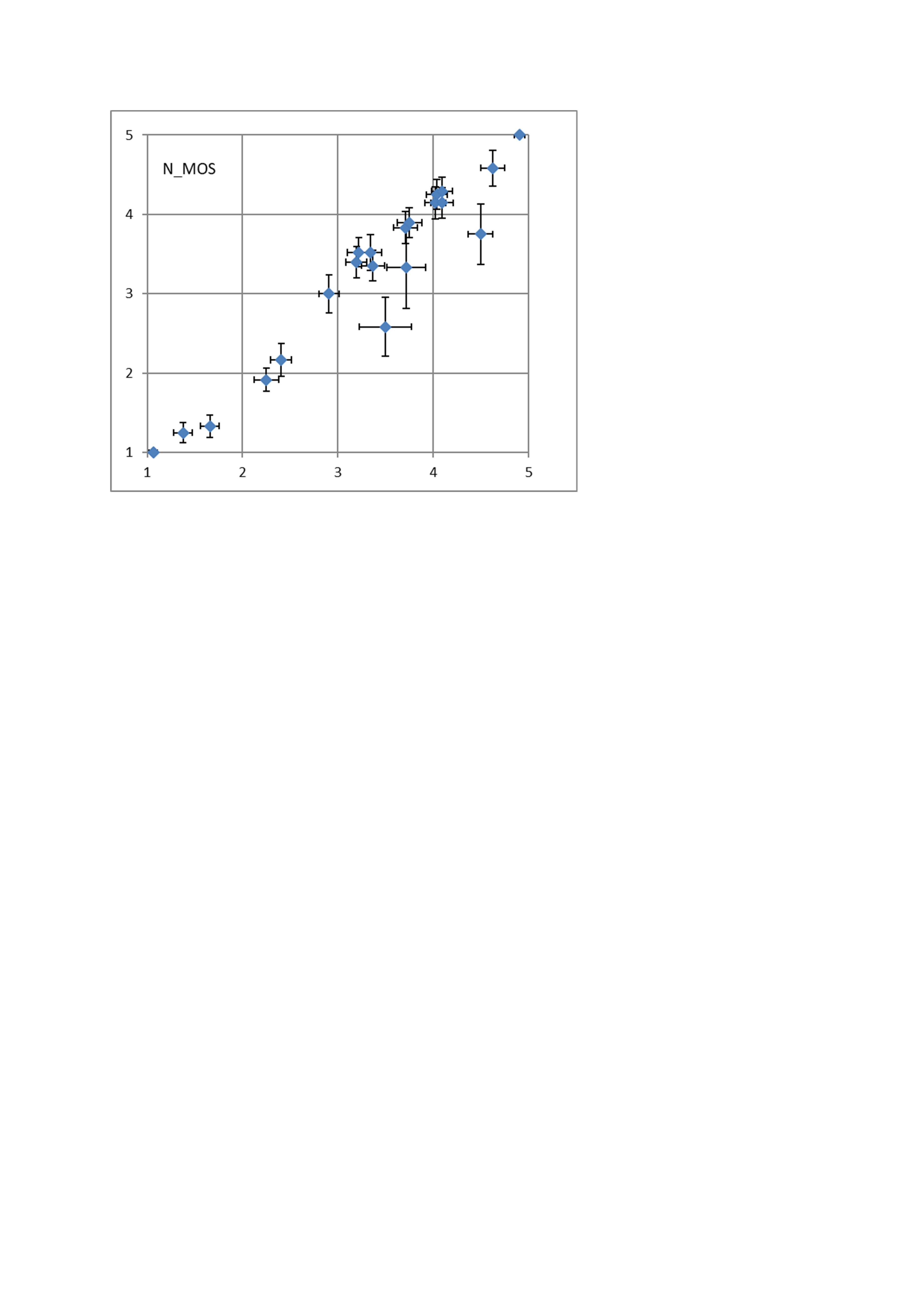

Supplement: S2 Fig — Noise annoyance MOS (N-MOS) of A and B tests. Both axes have the values of MOS (1–5). (TIF) [file pone.0199787.s002.tif]

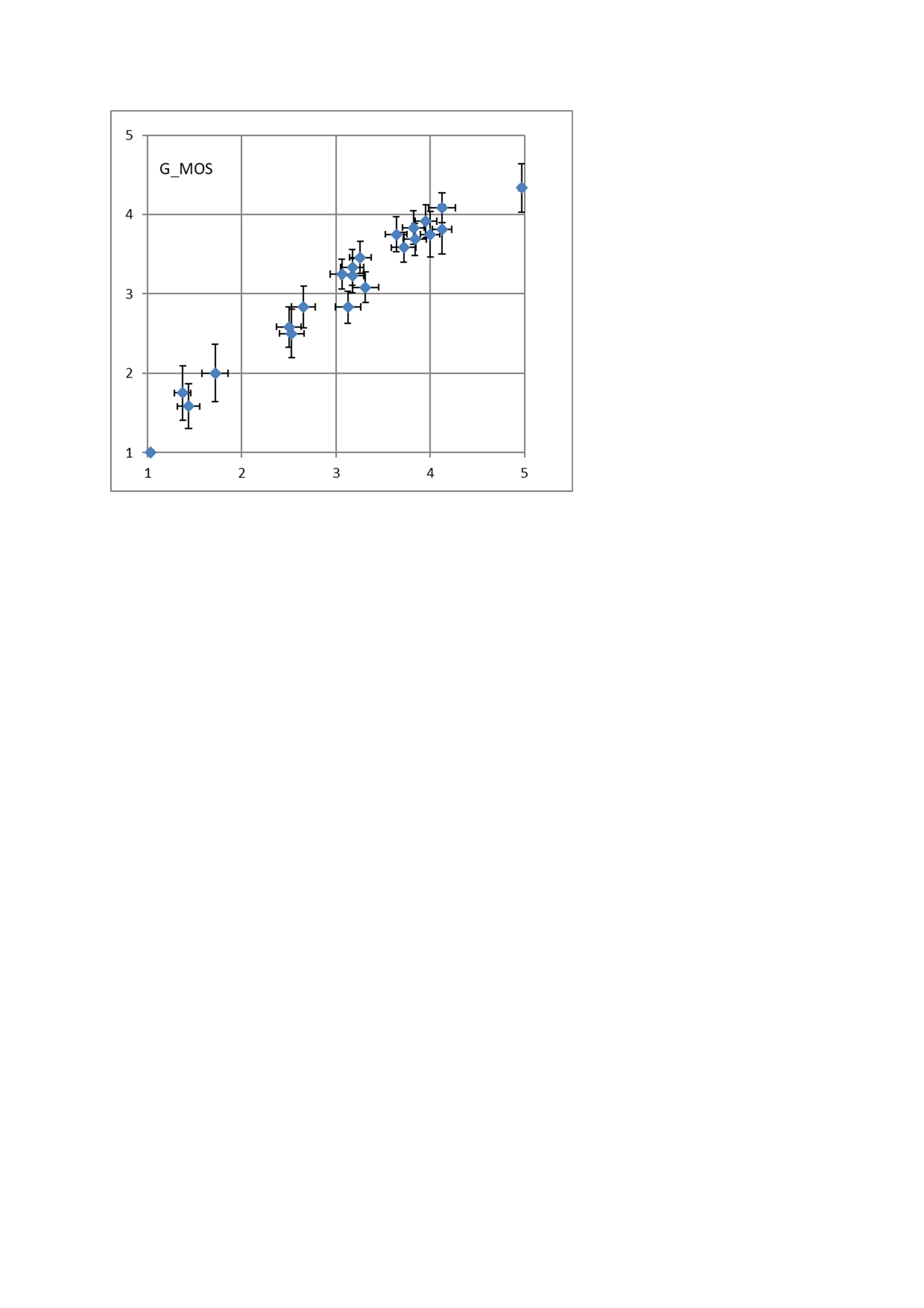

Supplement: S3 Fig — Overall quality MOS (G-MOS) of A and B test. Both axes have the values of MOS (1–5). (TIF) [file pone.0199787.s003.tif]
